# The challenge of timely diagnosis and management of acute leukemias and associated infections in Latin America

**DOI:** 10.1016/j.htct.2024.08.001

**Published:** 2024-08-09

**Authors:** Eduardo Magalhães Rego

**Affiliations:** a Instituto do Câncer do Estado de São Paulo (ICESP), São Paulo, SP, Brazil; b Instituto D´Or de Pesquisa e Ensino (IDOR), São Paulo, SP, Brazil; c Laboratório de Investigações Médicas 31, Faculdade de Medicina da Universidade de São Paulo (LIM 31 FM USP), São Paulo, SP, Brazil

The treatment of patients with acute leukemias (AL) is challenging not only because of the intrinsic aspects of the disease, such as the coexistence of multiple mechanisms of treatment resistance and the high frequency of clonal evolution, but also due to the significant resource demands placed on the health system for the prompt diagnosis, treatment, and management of adverse events related to the treatment. In this issue of our journal, two articles highlight the hurdles faced by doctors treating AL in Latin America.

Demichelis-Gómez et al.^1^ analyzed the time interval from the first symptom to treatment initiation in 188 Mexican patients with AL and found that the median interval was 31 days. Patients with acute lymphoblastic leukemia had longer intervals compared to those with acute promyelocytic leukemia (APL) and acute myeloid leukemia (AML), though all three groups had a median time longer than 20 days. The complete remission (CR) rate was 76.6 %, and 39 patients (20.7 %) died within 60 days of diagnosis (early death - ED). The main cause of ED was infection (64 %). Notably, across all treatment phases, 57 patients were suspected of having a fungal infection, with 22 (12.5 %) having confirmed fungal infections.

In another study, Nucci et al.^2^ analyzed 61 patients with AML treated at a reference university hospital in Brazil. The CR rate after the first course of intensive chemotherapy was 52.4 %. The authors detailed the adverse events observed during 67 cycles of high-dose cytarabine used as consolidation treatment for 28 patients. Febrile neutropenia occurred in 38 of these cycles (56.7 %), bacteremia in 13 cycles (34.2 %), and there were two cases of invasive fungal disease (IFD).

There is a paucity of studies reporting the time interval from symptom onset to diagnosis or treatment of acute leukemias. The International Consortium on Acute Leukemias reported that the interval between symptom onset and diagnosis of APL exceeded 10 days in 44.7 % of 806 cases treated in Brazil, Chile, Peru, Paraguay, and Uruguay. Sekeres et al.^3^ analyzed 1317 patients with AML from the Cleveland Clinic and M.D. Anderson Cancer Center databases (1994–2005) and found that longer intervals between diagnosis and treatment were associated with worse CR rates and overall survival in patients younger than 60, but not in older patients. In contrast, Rollig et al.^4^ found no association between the diagnosis-to-treatment interval and survival in a large dataset of 2263 AML patients from the German Study Alliance Leukemia-Acute Myeloid Leukemia (SAL-AML) registry.

The direct comparison between the data from Demichelis-Gómez et al.^1^. and those from Europe or the US is challenging due to differences in variables and methods. However, the data suggest that AL diagnosis and treatment initiation take longer in Latin America.

Regarding the frequency of infections during intensive chemotherapy in AL patients treated in Latin America, the results from Demichelis-Gómez et al.^1^ and Nucci et al.^2^ indicate that bacterial infections, rather than IFD, are particularly concerning. The former has been reported as a primary cause of ED in various studies from the region. However, recent studies show that the frequency of IFD in Latin American centers is similar to that reported in Europe. For example, Rodríguez-Veiga et al.^5^ found that out of 589 intensive chemotherapy episodes in 285 AML patients at a Spanish referral center, there were 56 (10 %) IFD episodes. According to the EORTC 2008 criteria, IFD was classified as possible (*n* = 29; 52 %), probable (*n* = 17; 30 %), and proven (*n* = 10; 18 %). The possible/probable/proven IFD rate was significantly lower during consolidation courses based on high doses of cytarabine compared to anthracycline-containing chemotherapy courses (2% vs. 11 %).

In summary, these two manuscripts emphasize that to improve patient outcomes in Latin America requires better access to specialized hematological care, improved diagnosis and management of infectious complications.

## Conflicts of interest

The author declares no conflicts of interest.
